# Impact of the 2011 Tohoku Earthquake on the species diversity of rocky intertidal sessile assemblages

**DOI:** 10.1002/ece3.70293

**Published:** 2024-09-16

**Authors:** Yuan Yao, Jingru You, Ken Ishida, Takashi Noda

**Affiliations:** ^1^ Graduate School of Environmental Science Hokkaido University Sapporo Japan; ^2^ Marine Biodiversity and Environmental Assessment Research Center Research Institute for Global Change, Japan Agency for Marine‐Earth Science and Technology (JAMSTEC) Yokosuka Japan; ^3^ Faculty of Environmental Earth Science Hokkaido University Sapporo Japan

**Keywords:** cataclysmic disturbance, diversity recovery, Pacific coast, sessile benthos, spatial heterogeneity, subsidence

## Abstract

The impacts of large‐scale disturbance events on the species diversity of rocky intertidal sessile assemblages across multiple spatial scales are not well understood. To evaluate the influence of the 2011 Tohoku Earthquake on alpha and beta diversities of rocky intertidal sessile assemblages, we surveyed sessile assemblages in the mid‐shore zone from 2011 to 2019 and compared the data with those collected from 2003 to 2010 before the earthquake at the same region. The census was conducted across 22 study plots on five rocky shores along 30 km of the Sanriku Coast of Japan, which is located 150–160 km north–northwest of the earthquake epicenter. Alpha diversity was measured with three Hill numbers (*H*_0_, *H*_1_, and *H*_2_), which represent the number of equally common species that would exist in a community with the same diversity as the sampled community, with higher values of the subscript indicating more weight placed on abundant species. Beta diversity was measured with two metrics (*BD*_total_ at two spatial scales). Values were compared between the post‐earthquake period (2011–2019) and the pre‐earthquake period (2003–2010). The results show that the Tohoku Earthquake significantly altered the species diversity of intertidal sessile assemblages across multiple spatial scales. All diversity metrics obtained at multiple spatial scales (i.e., alpha diversities: *H*_0_, *H*_1_, and *H*_2_; beta diversities: *BD*_*t*otal_ at the shore and regional scales) decreased immediately after the earthquake and then increased in subsequent years. At 2 years after the earthquake, *H*_0_ recovered to within the range of pre‐earthquake values and *H*_1_ and *H*_2_ became significantly higher than pre‐earthquake values. Most metrics of alpha and beta diversities recovered to pre‐earthquake levels after several years, but regional *BD*_total_ remained low for a longer period.

## Graphical abstract

The 2011 Tohoku Earthquake in Japan had a serious impact on rocky intertidal community. We assessed alpha diversity, beta diversity, and their effect sizes during the pre‐earthquake period (2003–2010) and post‐earthquake period (2011–2019) at local scale and regional scale. Both alpha diversity and beta diversity significantly declined after the earthquake at two scales and returned to pre‐earthquake levels within 3 years after the earthquake except for beta diversity at regional scale.

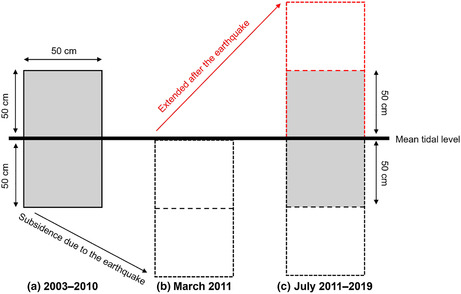

## 1 INTRODUCTION

Understanding the impacts of natural disturbances on biological communities is a central task of ecologists because disturbance is a major driver of community dynamics and species diversity (Connell, 1978; Huston, 1979; Sousa, 1984) and is particularly important for ecosystem functioning (e.g., Lohbeck et al., 2016; Tilman, 1997). Natural disturbances can affect species diversity in various ways. At the local scale, species diversity decreases immediately after a disturbance and then subsequently increases because disturbance indirectly favors inferior competitors by increasing resource availability through the mortality of dominant species (Fuentes & Brante, 2014; Huston, 1995). Therefore, diversity at the local scale should be highest at intermediate disturbance frequency and intensity due to prevention of competitive exclusion and promotion of spatiotemporal heterogeneity (Connell, 1978; England et al. 2008; Huston, 1979; Menge & Sutherland, 1987; Sousa, 1984; Whitaker et al., 2023). However, the effect of disturbance on species diversity at the regional scale (i.e., at the metacommunity level) is less clear, because disturbances can affect the rates of both species extinction and immigration in a complex way via alterations of resource availability, larvae supply, priority effects of foundation species, and environmental heterogeneity at the landscape level (Chase et al., 2005; Holt et al., 2005; Huston, 1995; Menge, 2000; Rosenzweig, 1995; Underwood, 2000; Weidlich et al., 2021; Whitaker et al., 2023). Furthermore, the effects of disturbances on species diversity vary at both local and regional scales depending on the specific disturbance agents (Huston, 1995; Mackey & Currie, 2001; Sousa, 2001).

Earthquakes and associated tsunamis have severely impacted coastal benthic communities around the world. The uplift induced by earthquake was the main cause of mortality of sessile organisms in rocky intertidal habitats for both Chilean earthquake of 3 March 1985 and Chilean mega‐earthquake of 27 February 2010 (Castilla, 1988; Castilla et al., 2010; Castilla & Oliva, 1990; Ortlieb et al., 1996). The abundance and biomass of macrobenthos in a soft‐bottom community significantly increased after the 2007 tsunami and earthquake in Paracas Bay, Peru, and they remained higher 4 months later (Lomovasky et al., 2011). Similarly, 3 years after the mega‐thrust earthquake in Maule, Chile, in 2010, the density, biomass, and diversity of epibenthic macrofaunal community at different locations were structured differently, with apparent directionality, and there was spatial homogenization of the community (Hernández‐Miranda et al., 2014). Another tsunami that hit Tetepare Island in 2010 reduced the cover and diversity of the seagrass community, and recovery to pre‐tsunami levels did not occur within the next 8 years (Moseby et al., 2020). The main causes of these tsunami‐induced effects are increased sedimentation and uplift or subsidence of the seafloor (Chunga‐Llauce & Pacheco, 2021). The speed of recovery from the earthquake may usually be affected by colonization ability of species (Paine & Levin, 1981; Wootton, 1993), larvae supply of the metapopulation (Kinlan & Gaines, 2003), and the indirect influence of the earthquake and associated tsunami via other species.

The influence of earthquakes and subsequent tsunamis on species diversity should be elucidated at multiple spatial scales. The influence of the 2010 Maule mega‐thrust earthquake on the species richness and abundance of sandy intertidal communities differed significantly among locations (Sepúlveda & Valdivia, 2016, 2017), suggesting spatial heterogeneity and a local specificity of impacts. This phenomenon might be caused by differences in species characteristics (e.g., high mobility and other species' adaptations) and habitat characteristics (e.g., the dynamic environmental conditions of sandy beaches and hydrological characteristics such as upwelling) (Jaramillo et al., 2003; Loreau et al., 2003; Menge & Menge, 2019; Shanks & Morgan, 2018; Valdivia et al., 2014). However, the effects of earthquakes and tsunamis on species diversity across spatial scales generally remain unknown due to a lack of systematic pre‐earthquake baseline datasets (Sepúlveda & Valdivia, 2016; Underwood, 2000). A case study that has a hierarchical spatial structure and experienced a large‐scale disturbance with consistent baseline data is the key to understanding the influence of earthquakes and tsunamis on species diversity across spatial scales.

The 2011 Tohoku Earthquake, which had a moment magnitude (Mw) of 9.0, triggered a huge tsunami with run‐up heights exceeding 30 m and caused 35–70 cm of subsidence throughout the Pacific coastline of the Tohoku region of northern Japan (Lay & Kanamori, 2011; Shimada, 2016; Tajima et al., 2013). The earthquake heavily altered the distribution and abundance of rocky intertidal sessile organisms (Iwasaki et al., 2016; Iwasaki & Noda, 2018; Kanamori et al., 2020; Noda, Iwasaki, & Fukaya, 2016a, 2016b). However, the impact of mega‐earthquakes on the species diversity of intertidal sessile assemblages has not been fully evaluated. The same earthquake significantly decreased the species richness, evenness, and Shannon–Wiener diversity of macrozoobenthic assemblages in Gamo Lagoon, Japan (Kanaya et al., 2015). Although studies have examined the effects of various disturbance agents such as storms, ice scars, severe winters, and earthquakes on the species diversity of intertidal sessile assemblages (Kunze et al., 2021; Sousa, 1984, 2001), these effects have rarely been examined across multiple spatial scales. Thus, to understand how large‐scale disturbance events affect the species diversity of intertidal sessile assemblages across spatial scales, a sample with the geographic consistency of the disturbance is a better comparison across multiple spatial scales.

We addressed the following three hypotheses about the course and recovery status of alpha and beta diversities of rocky intertidal sessile assemblages after the 2011 Tohoku Earthquake. First, we hypothesize that alpha diversity would have decreased immediately after the earthquake and then changed to pre‐earthquake levels because the tsunami associated with the earthquake caused mortality of dominant species (Iwasaki & Noda, 2018) but would not affect the recovery led by recruitment of larvae and propagules from shoreward transport (Addessi, 1994; Menge & Sutherland, 1987). Second, species dominance (or evenness) would have changed after the earthquake because species responses (resistance to and recovery from the earthquake) vary with species traits (Kanamori et al., 2020). Third, beta diversity would have increased after the earthquake due to an increase in the patchiness of sessile organisms' distributions (Castilla, 1988; Castilla et al., 2010; Castilla & Oliva, 1990; Ortlieb et al., 1996) caused by variations in coastal topography and geology that affected the force of the tsunami impact and susceptibility to wave action (Mori et al., 2011; Wijetunge, 2006).

To test the three hypotheses, we censused sessile assemblages from 2003 to 2019 in the mid‐shore zone on five shores along 30 km of the Sanriku Coast. Then, we compared three metrics of alpha diversity (*H*_0_, *H*_1_, and *H*_2_) and two metrics of beta diversity (*BD*_total_ at two spatial scales) for 2011–2019 with corresponding metrics for the pre‐earthquake period (2003–2010).

## 2 MATERIALS AND METHODS

### 2.1 Census design

**FIGURE 1:**
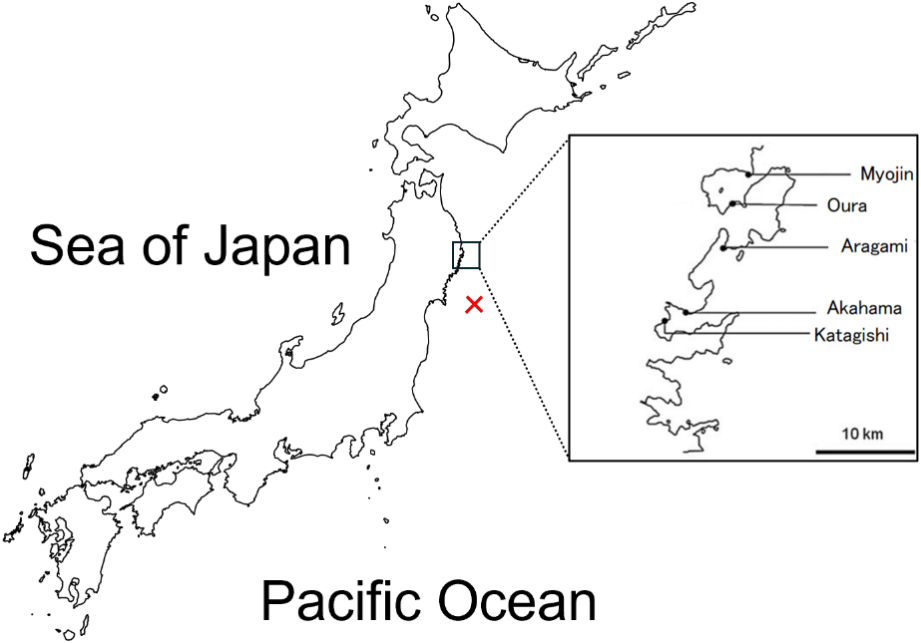
Map of study sites. Five rocky shores (filled circles) were chosen for the census to evaluate the effect of the 2011 Tohoku Earthquake on the alpha and beta diversities of rocky intertidal sessile assemblages along the Sanriku Coast of Japan in the Pacific Ocean. The red cross indicates the epicenter of the 2011 Tohoku Earthquake.

Rocky intertidal sessile assemblages were monitored at 22 plots on five rocky shores (Figure 1) located 2.6–7.9 km apart from each other along the Pacific coast of Japan. The study shores are located 150–160 km north–northwest of the epicenter (38°06′12.0″ N, 142°51′36.2″ E) of the 2011 Tohoku Earthquake. The detailed descriptions of the biogeographic features of the same census area were reported by Okuda et al. (2004), Nakaoka et al. (2006), Fukaya et al. (2010), and Ishida et al. (2021).

Within each shore, three to five plots were haphazardly chosen from semi‐exposed locations, with distances between neighboring plots ranging from 8 to 209 m (mean ± SD = 59 ± 70 m). Each plot was established on steep rock walls and spanned 50 cm wide by 100 cm in vertical extent, and the mean tidal level corresponded to the vertical mid‐point.

**FIGURE 2:**
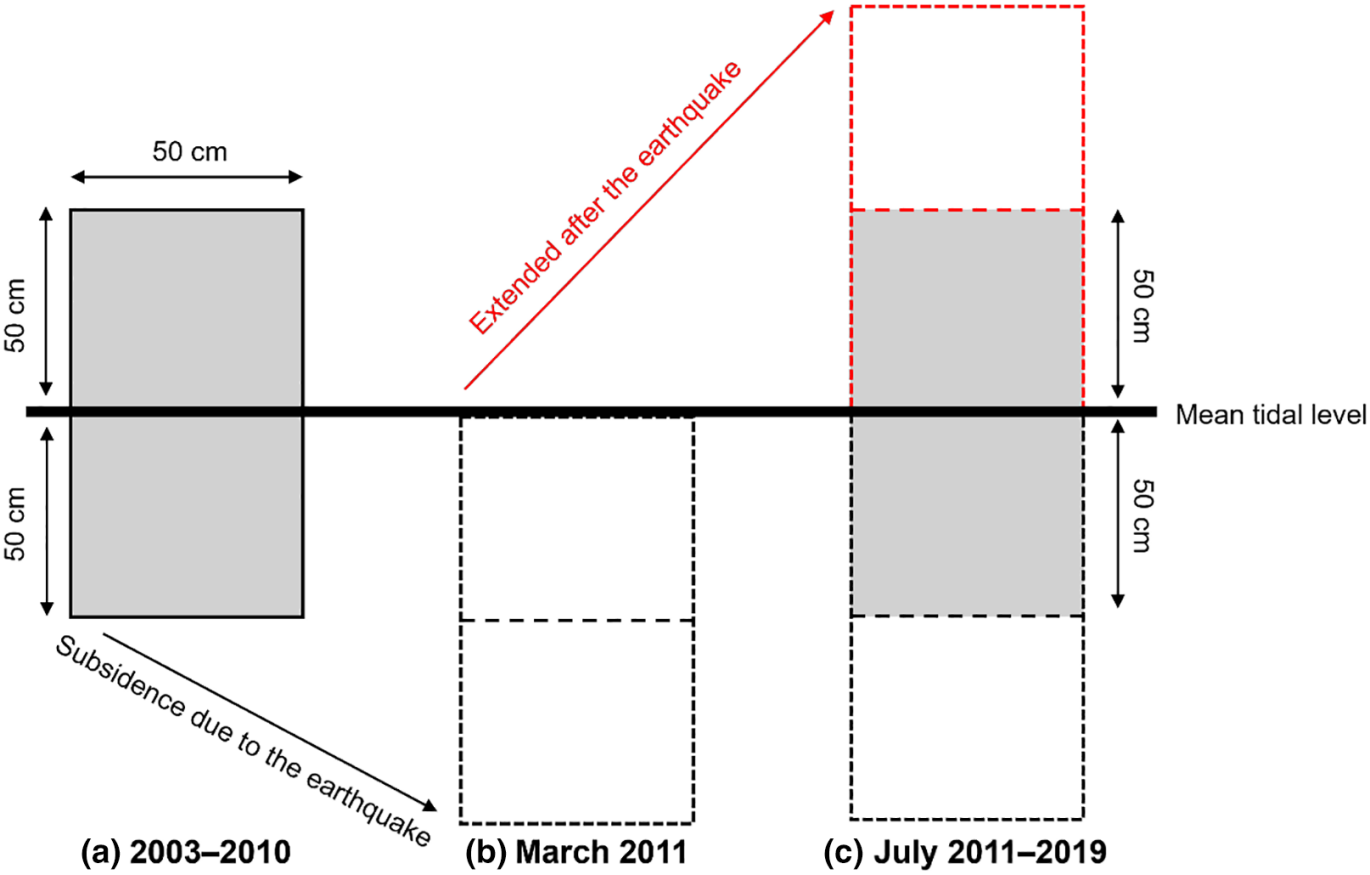
Conceptual diagram of census plot before and after the 2011 Tohoku Earthquake. Plots were established in 2003 (a) and experienced 50–60 cm of subsidence after the earthquake in March 2011 (b). We raised the upper margins of the plots by 100 cm in July 2011 (c). The thick solid black line indicates mean tidal level. The column bounded by solid black lines represents the original position of the plots established in 2003. The column bounded by dotted black lines represents the position of the control plots after the earthquake. The column bounded by dotted red and black lines represents the position of the extended plots after the earthquake. The gray shading shows the area that was investigated in this study.

The upper margin and lower margin of each plot was extended respectively by 50 cm in July 2011 (Figure 2), because the study area experienced subsidence during the 2011 Tohoku Earthquake; vertical subsidence was 50 cm at four of the five shores (Myojin, Oura, Aragami, and Katagishi) and 60 cm at Akahama (Noda, Iwasaki, & Fukaya, 2016a). Consequently, the vertical observation range was 200 cm after the earthquake at each plot.

**FIGURE 3:**
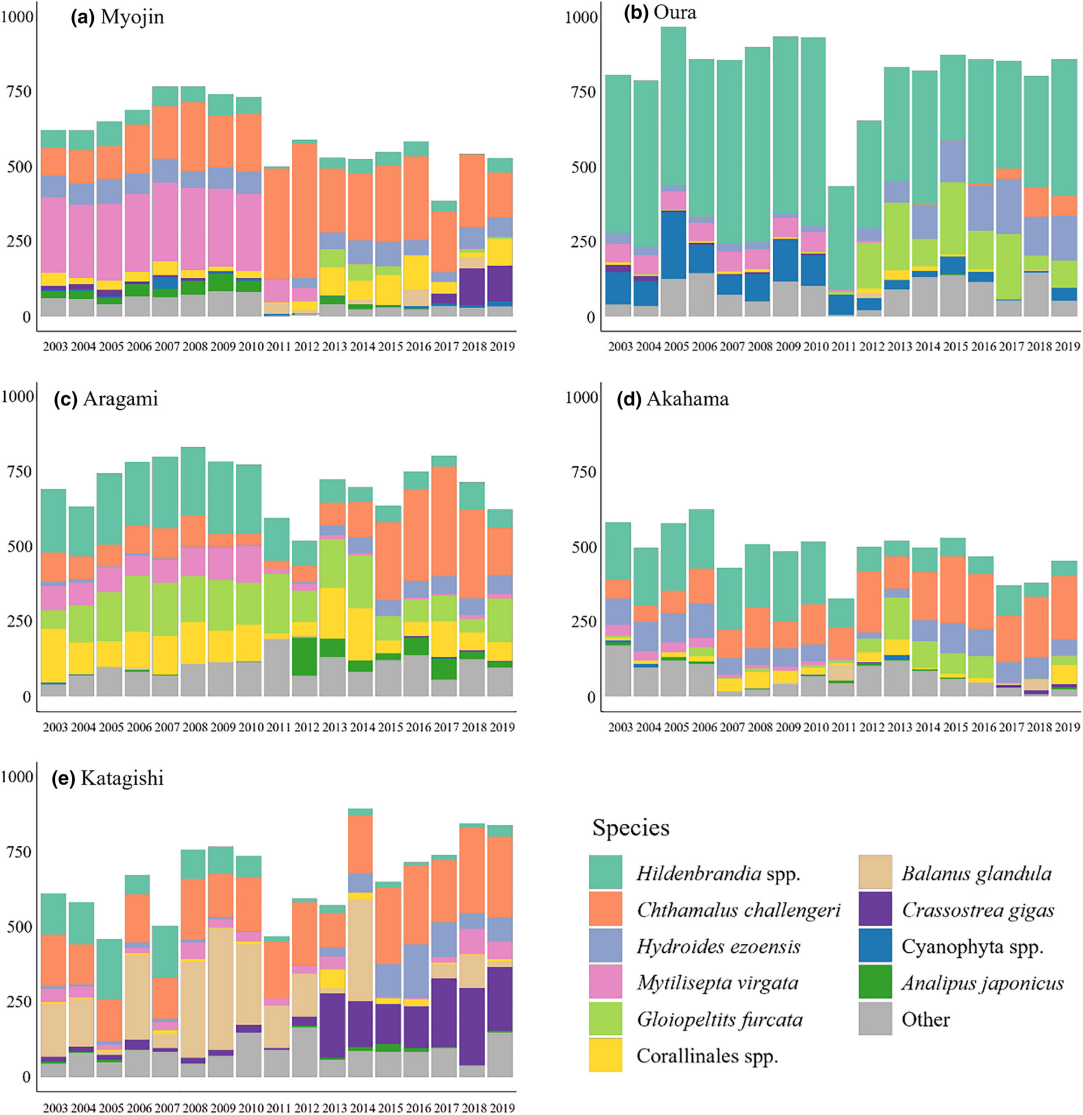
Interannual changes in relative abundance of the 10 most abundant species at the shore scale. The relative abundance is calculated by the sum of grid number occupied by each species in each shore. A total of 95 species were recorded during the survey period in all census plots. The top 10 species account for 88.32% of total relative abundance during census period. The order from top to bottom in the columnar accumulation diagram is arranged in order of total species coverage at the regional scale from most to least. Remaining species not in the top 10 were grouped as “other.”

For each plot, the estimates of the coverage of each sessile species were obtained from the same elevational range (1 m) of the plot before and after the earthquake such that the census area corresponded to the mean tidal level ± 50 cm (Figure 2) by using a grid overlain on the plot with 200 observation points inside the plot at evenly spaced intervals (5‐cm intervals in both the vertical and the horizontal directions). Each sessile organism occupying a grid point was identified and recorded, and the total number of grid points for each sessile organism was determined in each census (time series relative abundance of dominant species is shown in Figure 3). This census was carried out in July during low tide from 2003 to 2019.

### 2.2 Data analysis

#### 2.2.1 Diversity estimation

Alpha diversities for each plot in each year were calculated as Hill's diversity numbers (*H*_*q*_) (Jost, 2006), which represent the number of equally common species that would exist in a community with the same value as the sampled community (i.e., the effective number of species), with higher values of *q* indicating more weight placed on abundant species (Takada, 2015):

$$H_{q}={\left(\sum_{i=1}^{S}p_{i}^{q}\right)}^{1/\left(1-q\right)}$$

$$H_{0}=\sum_{i=1}^{S}p_{i}^{0}=S$$

$$H_{1}=\lim\left(q\to 1\right)H_{q}=\exp\left(-\sum_{i=1}^{S}p_{i}\ln\left(p_{i}\right)\right)$$

$$H_{2}=1/\sum_{i=1}^{S}p_{i}^{2}$$

where *S* is the total number of species and *p*_*i*_ is the relative abundance of the *i*th species. *H*_0_ represents the total number of species (species richness); *H*_1_ considers both richness and evenness, giving more weight to common species, which is the exponential of the Shannon entropy, and *H*_2_ emphasizes the most abundant species, giving more weight to species with higher abundances, which is the inverse of Simpson concentration index.

*BD*_total_ (Legendre & De Cáceres, 2013) was used as the measure of beta diversity, which was calculated as the total variation of the community for each year as follows:

$${\mathrm{BD}}_{\text{total}}=\frac{\Sigma_{i=1}^{S}\Sigma_{j=1}^{n}{\left(p_{\mathrm{ij}}-\bar{p_{j}}\right)}^{2}}{\left(n-1\right)}$$

where *S* and *n* are total numbers of species and plots, respectively. $p_{\mathrm{ij}}$ is abundance of the *i*th species in plot *j*, and $\bar{p_{j}}$ is the mean value of the corresponding *i*th species. Values of ${\mathrm{BD}}_{\text{total}}$ were obtained at both shore scale and regional scale (across all shores).

### 2.3 Statistical analyses

To evaluate the magnitude of difference in each diversity component ($H_{0}$, $H_{1}$, $H_{2}$, and ${\mathrm{BD}}_{\text{total}}$) between each year of the post‐earthquake period (2011–2019) and the mean of the pre‐earthquake period (2003–2010), the effect size of single‐subject data and its 95% confidence interval (CI) were obtained as follows (Busk & Serlin, 1992; Hurwitz et al., 2015; Olive & Smith, 2005; Petursdottir et al., 2009):

$${\mathrm{ES}}_{i}=\frac{x_{t}-\bar{x_{\text{before}}}}{{\mathrm{SD}}_{\text{before}}}$$

where $x_{t}$ and $\bar{x_{\text{before}}}$ represent the value of a diversity component in post‐earthquake year *t* and the mean value in the pre‐earthquake period (2003–2010), respectively. ${\mathrm{SD}}_{\text{before}}$ denotes the standard deviation of values in the pre‐earthquake period. The ES_*i*_ values of *H*_0_, *H*_1_, and *H*_2_ were obtained for each census plot, and the ES_*i*_ of *BD*_total_ was obtained at both the shore and regional scale.

We used Cohen's standardized measure of difference to assess the significance of effect size. An absolute value less than 0.3, around 0.5, and greater than 0.8 is considered to represent small, medium, and large effects, respectively (Cohen et al., 1988). If the 95% CI does not cross the zero line, this indicates that the diversity component for post‐earthquake year *t* was significantly different from that for the pre‐earthquake period. Alternatively, if there is no confidence interval for the estimated effect size, an effect size greater than 1.96 in absolute value suggests that the diversity component for post‐earthquake year *t* was significantly different from that for the pre‐earthquake period (Borenstein et al., 2021; McLeod et al., 2016). This threshold of 1.96 corresponds to the two‐tailed significance level of *p* < .05 commonly used in hypothesis testing. It is derived from the standard normal distribution and serves as a critical value for determining statistical significance.

## 3 RESULTS

### 3.1 Alpha diversity

#### 3.1.1 *H*_0_

**FIGURE 4:**
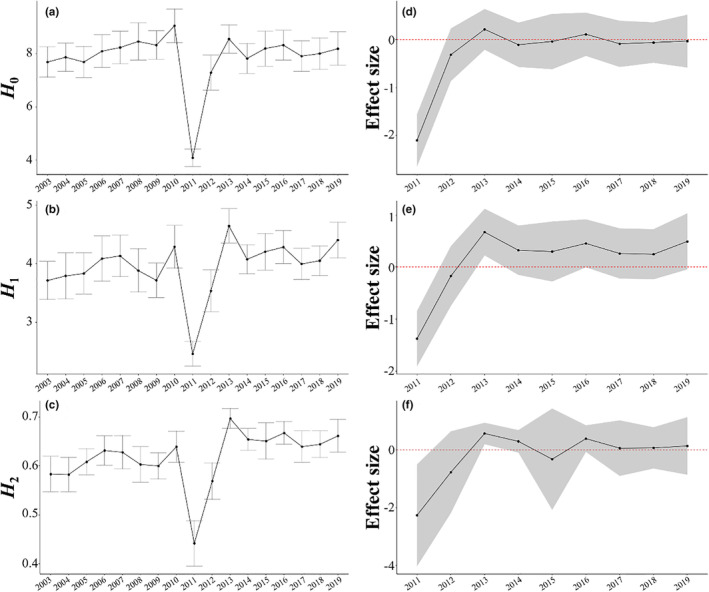
Times series of alpha diversity at the shore scale. (a–c) Time series of mean *H*_0_, *H*_1_ and *H*_2_ for each plot during the pre‐earthquake period (2003–2010) and post‐earthquake period (2011–2019), respectively. Error bars show SE. (d–f) Time series of mean effect size of *H*_0_, *H*_1_ and *H*_2_for each plot during the post‐earthquake period, respectively. The gray‐shaded area shows the 95% confidence interval, and the red horizontal dashed lines indicate an effect size of 0. If the red horizontal dashed line is included in 95% confidence interval (gray‐shaded area), it means that there has been no significant change from pre‐earthquake period.

Before the earthquake (2003–2010), the mean value of *H*_0_ varied within a small range (from 6 to 8; Figure 4a, Table 1). In 2011, when the earthquake occurred, the 95% CI of its effect size did not cross the zero line and the mean value of *H*_0_ decreased to nearly 4 (Figure 4d, Table 1), which was significantly lower than the level before the earthquake. Subsequently, the mean value of *H*_0_ increased and recovered to nearly pre‐earthquake levels after 2 years (2013; Figure 4a,d).

**TABLE 1:** Time series of diversity index at shore scale and regional scale. At shore scale, the mean and standard error of *H*_0_, *H*_1_, *H*_2_ and *BD*_total_ at each of five shores is shown. At regional scale, the value of *BD*_total_ is shown.

Shore scale									Regional scale
Year	*H*_0_		*H*_1_		*H*_2_		*BD*_total_		*BD*_total_
	Mean	SE	Mean	SE	Mean	SE	Mean	SE	
2003	7.68	0.57	3.71	0.32	0.58	0.04	2921.24	853.05	3076.93
2004	7.86	0.53	3.79	0.39	0.58	0.04	2506.18	651.51	2646.06
2005	7.68	0.59	3.83	0.35	0.61	0.03	2550.43	653.16	2655.03
2006	8.09	0.62	4.09	0.38	0.63	0.03	2462.23	574.51	2576.59
2007	8.23	0.61	4.13	0.36	0.63	0.03	2032.02	855.36	2146.58
2008	8.45	0.70	3.88	0.37	0.60	0.04	2632.36	952.04	2798.05
2009	8.32	0.54	3.72	0.29	0.60	0.03	2667.85	870.73	2862.54
2010	9.05	0.63	4.29	0.37	0.64	0.03	2222.16	676.58	2340.24
2011	4.09	0.33	2.47	0.21	0.44	0.05	1844.47	1048.00	2011.12
2012	7.29	0.66	3.54	0.36	0.57	0.04	2015.31	215.20	2028.53
2013	8.55	0.53	4.64	0.29	0.70	0.02	1750.72	364.79	1794.32
2014	7.82	0.56	4.08	0.25	0.65	0.02	2098.69	221.64	2122.03
2015	8.19	0.66	4.20	0.31	0.65	0.04	1941.73	491.99	1905.95
2016	8.32	0.56	4.28	0.28	0.67	0.02	1721.96	403.02	1753.34
2017	7.90	0.57	4.00	0.26	0.64	0.03	1981.77	374.04	1995.89
2018	8.00	0.59	4.05	0.25	0.64	0.03	2017.16	462.23	2114.72
2019	8.18	0.63	4.40	0.31	0.66	0.03	2082.75	653.34	2220.75

#### 3.1.2 *H*_1_

Before the earthquake, the mean value of *H*_1_ varied within a small range (around 4; Figure 4b, Table 1). In 2011, when the earthquake occurred, the 95% CI of its effect size did not cross the zero line and the mean value of *H*_0_ decreased to 2.5 (Figure 4e, Table 1), which was significantly lower than the level before the earthquake. After the earthquake, the value of *H*_1_ increased rapidly until 2013 (Figure 4b); the mean value of *H*_1_ at 2 years after the earthquake (2013) was significantly higher than the level before the earthquake, and the 95% CI of its effect size did not cross the zero line (the lower bound of 95% CI was 0.226; Figure 4e, Table 1). Subsequently, the mean value of *H*_1_ returned to almost the same level as before the earthquake, and its 95% CI crossed the zero line (Figure 4b,e).

#### 3.1.3 *H*_2_

Before the earthquake, the mean value of *H*_2_ varied within a small range (around 0.6; Figure 4c, Table 1). In 2011, when the earthquake occurred, it decreased to 0.45, which was significantly lower than the level before the earthquake (Figure 4f, Table 1). After the earthquake, the value of *H*_2_ increased rapidly until 2013. The value of *H*_2_ at 2 years after the earthquake (2013) was significantly higher than the level before the earthquake (the lower bound of 95% CI was 0.191; Figure 4f). Subsequently, the value returned to almost the same level as before the earthquake, and its 95% CI crossed the zero line (Figure 4c,f).

### 3.2 Beta diversity

#### 3.2.1 *BD*_total_ at the shore scale

**FIGURE 5:**
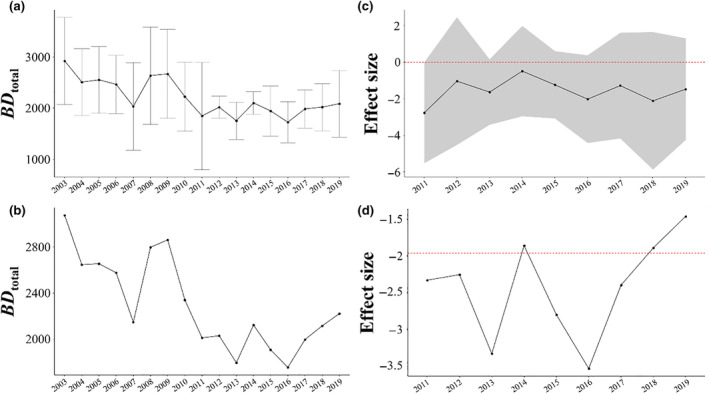
Times series of beta diversity at regional scale and shore scale. (a) Time series of mean *BD*_total_ at the shore scale during the pre‐earthquake period (2003–2010) and post‐earthquake period (2011–2019). Error bars show SE. (b) Time series of mean *BD*_total_ at the regional scale during the pre‐earthquake period and post‐earthquake period. (c) Time series of effect size of mean *BD*_total_ at the shore scale during the post‐earthquake period (2011–2019). The gray‐shaded area shows the 95% confidence interval, and the red horizontal dashed lines indicate an effect size of 0. If the red horizontal dashed line is included in 95% confidence interval (gray‐shaded area), it means that there has been no significant change from pre‐earthquake period. (d) Time series of effect size of *BD*_total_ at the regional scale during the post‐earthquake period. The red horizontal dashed line indicates an effect size of −1.96; values less than −1.96 indicate that the mean *BD*_total_ in that year falls outside the 95% prediction interval for the pre‐earthquake period.

Before the earthquake, the value of *BD*_total_ at the shore scale varied from 2500 to 3800 (Figure 5a, Table 1). In 2011, when the earthquake occurred, *BD*_total_ decreased to 2400, which was significantly lower than the level before the earthquake (Figure 5c, Table 1). After 2012, the value of *BD*_total_ at the shore scale remained relatively low until 2019, but any differences from the pre‐earthquake values were not significant (Figure 5a,c).

#### 3.2.2 *BD*_total_ at the regional scale

Before the earthquake, the value of *BD*_total_ at the regional scale varied from 2200 to 3200 (Figure 5b, Table 1). When the earthquake occurred, *BD*_total_ decreased to 2100, which was significantly lower than the value before the earthquake (Figure 5d, Table 1). Subsequently, the value of *BD*_total_ at the regional scale decreased continuously until 2013. Overall, *BD*_total_ at the regional scale was significantly lower for much of the post‐earthquake period (2011–2013 and 2015–2017) when compared with the pre‐earthquake period (Figure 5b,d).

## 4 DISCUSSION

The effects of earthquakes and subsequent tsunamis on the species diversity of coastal communities at different spatial scales have remained unclear until now. In this study, we assessed the effects of the 2011 Tohoku Earthquake on the species diversity of the rocky intertidal sessile community at both regional and local scales. Using the data of 22 study plots from 8 years after the earthquake and 8 years before the earthquake, the changes in alpha and beta diversities were estimated. The results show that all three components of alpha diversity (*H*_0_, *H*_1_, and *H*_2_) significantly decreased after the earthquake occurred and returned to almost the same level as before the earthquake by 3 years later. Moreover, the shore‐scale beta diversity (*BD*_total_) in the post‐earthquake period was only significantly lower than that for the pre‐earthquake period in 2011, whereas the *BD*_total_ at the regional scale was significantly lower than that for the pre‐earthquake period from 2011 to 2017, except in 2014.

### 4.1 Alpha diversity

The alpha diversity results are consistent with our first hypothesis that alpha diversity would decrease immediately after the earthquake and then subsequently recover toward the pre‐earthquake level: all metrics of alpha diversity (*H*_0_, *H*_1_, and *H*_2_) declined within the first year after the earthquake, and the values of *H*_1_ and *H*_2_ subsequently increased rapidly until 2013. The rapid declines of species richness caused by the 2011 Tohoku Earthquake were also reported in a macrozoobenthos community in Gamo Lagoon (Kanaya et al., 2015). Similarly, the earthquakes causing rapid declines in species richness (*H*_0_) of rocky intertidal community have also been reported in Kaikōura of November 2016, New Zealand (Schiel et al., 2019). The coseismic subsidence of 50–60 cm throughout the study area (Noda, Iwasaki, & Fukaya, 2016a) is considered a major cause of the decline of alpha diversity just after the earthquake because alpha diversity generally increased with elevation in the mid‐intertidal zone during the pre‐earthquake period (unpublished data) due to lower predator abundance and moderate desiccation. In addition, physical damage from the force of the tsunami, which caused significant population declines among several sessile organisms, such as mussels (Iwasaki et al., 2016), may also have contributed to the decline of alpha diversity immediately after the earthquake.

From 2011 to 2013, all metrics of alpha diversity increased, and by 2013 *H*_0_ had recovered to within the range of values observed before the earthquake, whereas *H*_1_ and *H*_2_ reached significantly higher values than those observed before the earthquake. This clearly indicates that the relative abundance of common species in 2013 was significantly higher than before the earthquake (Figure 3), supporting our second hypothesis that species dominance (or evenness) would change after the earthquake. This increase in the relative abundance of common species could have been associated with a temporary increase in free space availability caused by coseismic subsidence, because in the mid‐intertidal zone in our study area, the proportion of bare space on rock surface generally increased with elevation during the pre‐earthquake period (Sakaguchi, 2014). Moreover, a decline in the two competitive dominants, mussels (*Mytilisepta virgatus*) and the perennial crustose red alga *Hildenbrandia* spp. (Ishida et al., 2021; Miyamoto & Noda, 2004), during the first 3 years after the earthquake (Kanamori et al., 2020) might have contributed to an increase in the relative abundance of other species in the sessile assemblage in 2013.

### 4.2 Beta diversity

Based on the previous studies that assessed the impact of mega‐earthquakes on rocky intertidal communities on the Chilean coast (Castilla, 1988; Castilla et al., 2010; Castilla & Oliva, 1990; Ortlieb et al., 1996), we hypothesized that the beta diversity of rocky intertidal sessile assemblages on the Sanriku Coast would increase after the 2011 Tohoku Earthquake. Contrary to this prediction, *BD*_total_ tended to decline after the earthquake on both the shore and the regional scales. There are at least three non‐exclusive explanations for this result. First, the 2011 Tohoku Earthquake might have reduced, rather than increased, environmental heterogeneity (Castilla, 1988; Castilla et al., 2010; Castilla & Oliva, 1990; Ortlieb et al., 1996), which is a major cause of spatial patchiness in the distribution of rocky intertidal sessile organisms. In fact, landslides and slope failures, which are often associated with huge earthquakes, did not occur around our study plots (Noda et al., 2017). By contrast, the 50–60 cm of coseismic subsidence that did occur throughout the study area would have reduced the spatial heterogeneity of wave exposure by reducing the spatial variability (coefficient of variation) of water depth at the survey plots. Second, the tsunami might have enabled among‐shore transport of species that were unable to disperse between shores under normal hydrodynamic conditions before the earthquake. In fact, the tsunami and tsunami debris associated with the 2011 Tohoku Earthquake provided an opportunity for various sessile organisms to colonize areas outside of their original geographical distribution by transporting their propagules (Gewin, 2013). Third, in marine benthic habitats, early successional assemblages tend to be dominated by long‐distance dispersers, which show less spatially aggregated distributions (Foggo et al., 2007; Sahara et al., 2015) because their larval dispersal weakens the influence of any spatial heterogeneities in the adult distribution on the juvenile distribution pattern (Reed et al., 2000; Uriz et al., 1998). In fact, barnacles, such as *Chthamalus challengeri* and *Semibalanus cariosus*, whose abundance increased dramatically immediately after the earthquake (Kanamori et al., 2020; Noda, Iwasaki, & Fukaya, 2016b; Noda, Sakaguchi, et al., 2016), are capable of long‐distance dispersal over several kilometers as planktonic larvae and are fast growing (Kinlan & Gaines, 2003). Of the above three explanations for the decline of *BD*_total_ after the earthquake, the last two could also explain why regional *BD*_total_ remained low after the earthquake for a long period even after the quick recovery of *BD*_total_ at the shore scale.

Marine heatwaves (MHWs), which have become more frequent in recent years (Hobday et al., 2018; Oliver et al., 2018), often affect community dynamics of marine organisms (Ishida et al., 2023; Mieszkowska et al., 2021; Suryan et al., 2021; Weitzman et al., 2021; Ziegler et al., 2023). Indeed, it has been reported that the MHWs that occurred in Pacific coast of eastern Hokkaido from 2010 to 2016 (Miyama et al., 2021) affected the species composition and abundance of sessile organisms in rocky intertidal communities (Ishida et al., 2023). It is suspected that the decline in *BD*_total_ values observed in 2009 to 2010 confirmed in this study may be related to the occurrence of MHW. Therefore, we additionally estimated the effect of MHWs on *BD*_total_ by comparing the difference between *BD*_total_ value in years with and without MHWs. First, we investigated the occurrence of MHWs around Sanriku coast during the study period. The results showed that category I (moderate) MHWs (Hobday et al., 2016, 2018) occurred twice each in 2005 and 2007, and no MHW occurred in the period after the 2009 survey and before the 2010 survey (Tables S1 and S2). Furthermore, MHW occurred annually from 2011 to 2019, with a category II (strong) MHW (Hobday et al., 2016, 2018) in 2012 and category I (moderate) MHW in other years (Table S1). Second, we statistically estimated the effect of MHW on *BD*_total_. Before the 2011 Tohoku earthquake, because only the MHW that occurred in 2007 is included in the census data and the MHW in 2005 came after the census, we used ANOVA to compare the difference between *BD*_total_ in 2006 and in other years before the 2011, the difference between *BD*_total_ in 2007 and in other years before the 2011, and the difference between *BD*_total_ in 2006 and *BD*_total_ in 2007. The results showed that there was no significant difference in any of the above comparisons (Table S3). Third, we recalculated the effect size of *BD*_total_ with the baseline that excluded the data of 2006 and 2007. This recalculation did not change our results, indicating that the MHW years should be included in the baseline data for the pre‐earthquake years (Figure S1 in Data S1). Therefore, based on the above results, we think that the MHWs around Sanriku coast are weak and have no significant effect on BD_total_.

### 4.3 Ecological consequences of changes in species diversity

Although alpha and beta diversities at the shore‐scale returned to pre‐earthquake levels after experiencing rapid declines in 2011, the changes in species diversity and subsequent changes in community structure had several ecological consequences. First, the relative abundance of dominant species decreased after the disturbance (Figure 3), which could provide niche opportunities for invasion of rare species (Shea & Chesson, 2002). Second, the order of opportunistic species invasion may determine the process of succession, because priority effects of foundation species may alter the colonization, growth, or reproduction of other species that arrive later (Weidlich et al., 2021). Consequently, although the indices of species diversity returned to the pre‐earthquake level, the species composition and community structure changed obviously compared with that before the earthquake (Figure 3). Finally, drastic changes in relative abundance caused by external factors (i.e., disturbance) may also alter the direction and strength of species interactions, which may cause the changes in species distribution range. This is because the upper limits of rocky intertidal sessile species are determined by environmental stress and the lower limits by species interactions (Connell, 1961; Scrosati et al., 2011; Valdivia et al., 2011).

## 5 CONCLUSION

Despite the large amount of research conducted on the marine community ecology of rocky intertidal assemblages in recent decades (Hadiyanto et al., 2024; Ishida et al., 2021; Menge et al., 2022; Morgan, 2011; Scrosati et al., 2022), the effects of large‐scale disturbance events on the species diversity of this assemblage across multiple spatial scales remain poorly understood. The present study shows that the disturbance across spatial scales indeed altered the species diversity of intertidal sessile assemblages at multiple spatial scales. In the region 150–160 km north‐northwest of the epicenter of the 2011 Tohoku Earthquake, all of the diversity metrics (i.e., alpha diversities: *H*_0_, *H*_1_, and *H*_2_; beta diversities: *BD*_total_ at the shore and regional scales) examined from five shores located 2.6–7.9 km apart from each other immediately decreased after the earthquake and then increased in subsequent years. At 2 years after the earthquake (2013), *H*_0_ recovered to within the range of values observed before the earthquake, while *H*_1_ and *H*_2_ reached significantly higher values than those observed before the earthquake, indicating that species evenness became significantly lower than pre‐earthquake levels. At several years after the earthquake, although most metrics of alpha and beta diversities recovered toward pre‐earthquake levels, regional *BD*_total_ remained low for a prolonged period. The effects of disturbances at both local and regional scales on species diversity are likely to vary among disturbance agents (Huston, 1995; Mackey & Currie, 2001; Sousa, 2001). Although rocky intertidal habitats are vulnerable to various large‐scale disturbance agents, such as storms, ice scars, severe winters, and earthquakes (Kunze et al., 2021; Sousa, 1984, 2001), their consequences for metacommunity diversity remain poorly understood. Because the present study only revealed the long‐term pattern of changes in species diversity after the earthquake and associated tsunami, further research that elucidates the ecological consequences of changes in species diversity caused by large‐scale disasters and their mechanisms of recovery is eagerly awaited by ecologists and conservation biologists.

## AUTHOR CONTRIBUTIONS

**Yuan Yao:** Conceptualization (equal); data curation (equal); formal analysis (lead); investigation (equal); visualization (lead); writing – original draft (equal). **Jingru You:** Conceptualization (equal); data curation (equal); investigation (equal); writing – original draft (supporting). **Ken Ishida:** Data curation (equal); investigation (equal); writing – review and editing (equal). **Takashi Noda:** Conceptualization (equal); data curation (equal); funding acquisition (lead); investigation (equal); supervision (lead); writing – review and editing (lead).

## CONFLICT OF INTEREST STATEMENT

The authors declare no conflict of interest. The funders had no role in the design of the study; in the collection, analyses, or interpretation of data; in the writing of the manuscript; or in the decision to publish the results.

## Supporting information


Data S1:

## Data Availability

The data supporting the findings of this study are available in the Dryad Digital Repository, and can be accessed at https://doi.org/10.5061/dryad.vmcvdnd1r.
