# Supplementary material for: Impact of the 2011 Tohoku Earthquake on the species diversity of rocky intertidal sessile assemblages
Source: Ecol Evol. 2024 Sep 16;14(9):e70293. doi: 10.1002/ece3.70293 (PMC11405290; doi:10.1002/ece3.70293)
Supplement: Supplementary file 1 — Data S1: [file ECE3-14-e70293-s001.docx]

Table S1. The marine heatwave around Sanriku coast. For evaluating the marine heatwaves.

| category | duration | Start Date | Peak Date | End Date | Mean Intensity | Max Intensity | Variation of Intensity | Cumulative Intensity |
| --- | --- | --- | --- | --- | --- | --- | --- | --- |
| I Moderate | 17 | 2005/8/9 | 2005/8/18 | 2005/8/25 | 2.2486 | 2.451 | 0.171 | 38.2265 |
| I Moderate | 7 | 2005/8/30 | 2005/9/2 | 2005/9/5 | 1.935 | 2.0146 | 0.0767 | 13.5447 |
| I Moderate | 5 | 2007/6/24 | 2007/6/26 | 2007/6/28 | 2.0236 | 2.155 | 0.1173 | 10.1178 |
| I Moderate | 12 | 2007/10/31 | 2007/11/6 | 2007/11/11 | 1.5593 | 1.6677 | 0.0832 | 18.711 |
| I Moderate | 9 | 2010/7/21 | 2010/7/24 | 2010/7/29 | 2.1006 | 2.2376 | 0.1224 | 18.9056 |
| I Moderate | 50 | 2010/8/6 | 2010/8/30 | 2010/9/24 | 2.1225 | 2.6524 | 0.2706 | 106.1248 |
| I Moderate | 22 | 2010/10/18 | 2010/11/3 | 2010/11/8 | 1.5891 | 1.7859 | 0.1123 | 34.9609 |
| I Moderate | 5 | 2010/11/19 | 2010/11/21 | 2010/11/23 | 1.548 | 1.5838 | 0.032 | 7.7399 |
| I Moderate | 17 | 2011/7/2 | 2011/7/14 | 2011/7/18 | 2.5172 | 2.9861 | 0.3335 | 42.7929 |
| II Strong | 67 | 2012/8/22 | 2012/9/18 | 2012/10/27 | 2.6196 | 3.8495 | 0.7024 | 175.5122 |
| I Moderate | 7 | 2013/9/7 | 2013/9/10 | 2013/9/13 | 1.8828 | 2.0101 | 0.1164 | 13.1797 |
| I Moderate | 21 | 2014/6/18 | 2014/6/26 | 2014/7/8 | 2.9541 | 3.705 | 0.5314 | 62.0358 |
| I Moderate | 23 | 2015/7/22 | 2015/8/3 | 2015/8/13 | 2.9081 | 3.5848 | 0.5635 | 66.8853 |
| I Moderate | 25 | 2016/3/1 | 2016/3/7 | 2016/3/25 | 2.4143 | 2.6592 | 0.1851 | 60.3566 |
| I Moderate | 12 | 2016/9/28 | 2016/10/1 | 2016/10/9 | 1.6175 | 1.7277 | 0.0989 | 19.4103 |
| I Moderate | 10 | 2017/2/27 | 2017/3/4 | 2017/3/8 | 2.1906 | 2.4372 | 0.2177 | 21.9062 |
| I Moderate | 5 | 2018/2/7 | 2018/2/9 | 2018/2/11 | 1.7628 | 1.8104 | 0.0535 | 8.8142 |
| I Moderate | 13 | 2018/2/18 | 2018/2/25 | 2018/3/2 | 2.044 | 2.2876 | 0.2032 | 26.5724 |
| I Moderate | 12 | 2018/6/28 | 2018/7/3 | 2018/7/9 | 2.6375 | 3.2032 | 0.3907 | 31.6499 |
| I Moderate | 24 | 2018/7/13 | 2018/7/31 | 2018/8/5 | 2.1202 | 2.5109 | 0.2261 | 50.8837 |
| I Moderate | 6 | 2018/9/20 | 2018/9/23 | 2019/9/25 | 1.5908 | 1.6866 | 0.0885 | 9.545 |
| I Moderate | 18 | 2018/10/12 | 2018/10/22 | 2019/10/29 | 1.6467 | 1.9141 | 0.1784 | 29.6401 |
| I Moderate | 14 | 2019/7/29 | 2019/8/5 | 2019/8/11 | 2.6227 | 3.1207 | 0.4185 | 36.7182 |

Table S2. Survey schedule during the study period.

| Year | Start Date | End Date |
| --- | --- | --- |
| 2003 | 7/31 | 8/1 |
| 2004 | 7/15 | 7/20 |
| 2005 | 7/19 | 7/22 |
| 2006 | 8/7 | 8/10 |
| 2007 | 7/12 | 7/14 |
| 2008 | 7/29 | 7/30 |
| 2009 | 7/20 | 7/21 |
| 2010 | 7/9 | 7/11 |
| 2011 | 7/28 | 8/1 |
| 2012 | 7/15 | 7/17 |
| 2013 | 7/8 | 7/10 |
| 2014 | 7/24 | 7/26 |
| 2015 | 6/30 | 7/1 |
| 2016 | 7/3 | 7/5 |
| 2017 | 7/22 | 7/24 |
| 2018 | 7/11 | 7/13 |
| 2019 | 7/1 | 7/3 |

Table S3. The result of ANOVA for BD_total_ value comparisons between pre-earthquake years. Because the MHW occurred in 2005 (after the survey) and 2007 (before and during the survey), 2006 and 2007 were considered as MHW affected year.

| Year | | F value | P value |
| --- | --- | --- | --- |
| 2006 | 2003 | 0.199 | 0.667 |
|  | 2004 | 0.003 | 0.961 |
|  | 2005 | 0.001 | 0.922 |
|  | 2008 | 0.023 | 0.882 |
|  | 2009 | 0.039 | 0.849 |
|  | 2010 | 0.073 | 0.794 |
| 2007 | 2003 | 0.542 | 0.483 |
|  | 2004 | 0.194 | 0.671 |
|  | 2005 | 0.232 | 0.643 |
|  | 2008 | 0.22 | 0.652 |
|  | 2009 | 0.271 | 0.617 |
|  | 2010 | 0.03 | 0.866 |
| 2006 | 2007 | 0.174 | 0.687 |


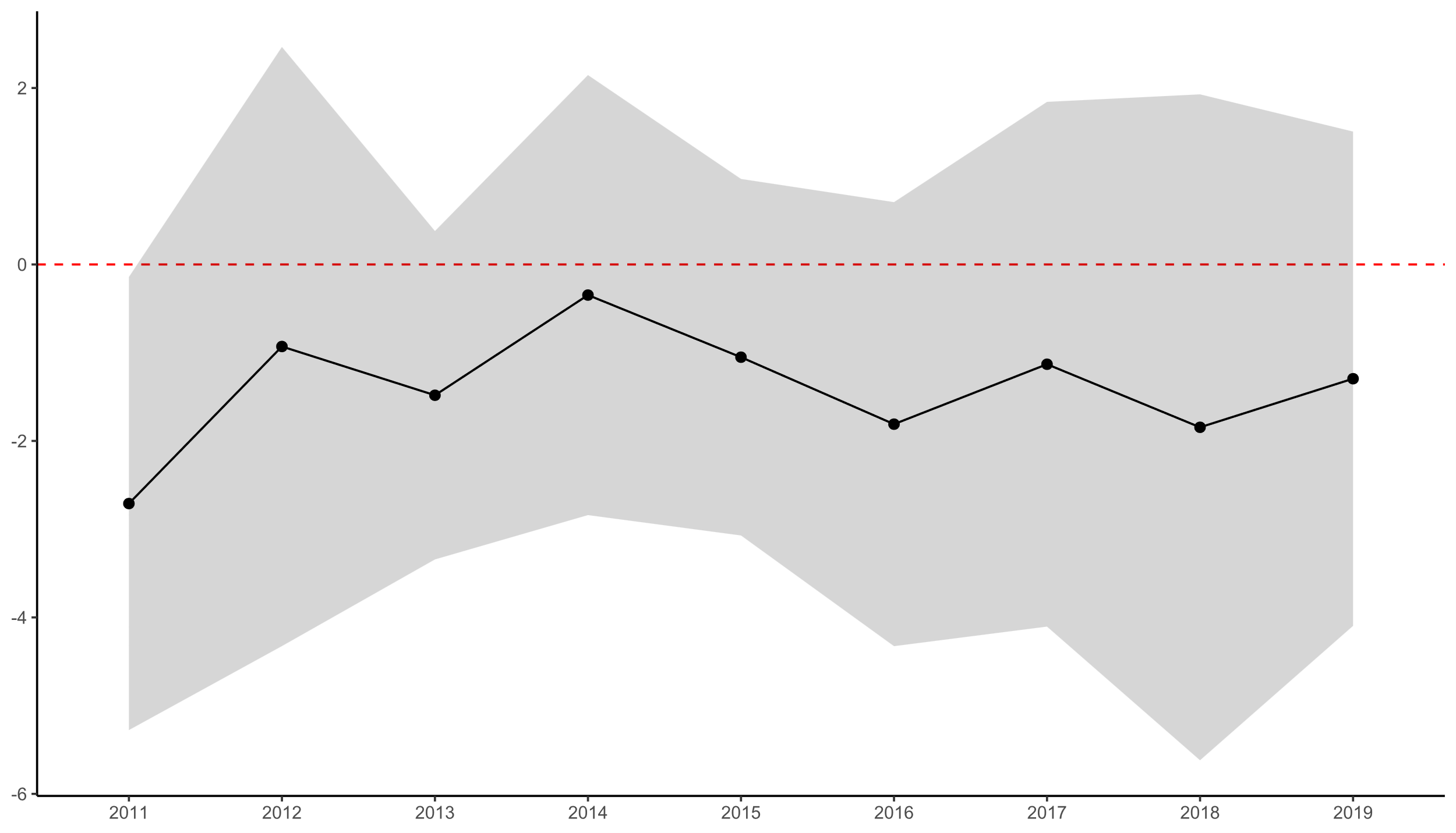


Fig.S1 Time series of effect size of mean *BD*_total_ at the shore scale during the post-earthquake period (2011–2019). The grey-shaded area shows the 95% confidence interval, and the red horizontal dashed lines indicate an effect size of 0. If the red horizontal dashed line is included in 95% confidence interval (grey-shaded area), it means that there has been no significant change from pre-earthquake period.
